# Photoreceptor Inner and Outer Segment Junction Reflectivity after Vitrectomy for Macula-Off Rhegmatogenous Retinal Detachment

**DOI:** 10.1155/2015/451408

**Published:** 2015-10-22

**Authors:** Jakub J. Kaluzny, Bartosz L. Sikorski, Grzegorz Czajkowski, Mateusz Burduk, Bartlomiej J. Kaluzny, Joanna Stafiej, Grazyna Malukiewicz

**Affiliations:** ^1^Department of Public Health, Collegium Medicum, Nicolaus Copernicus University, Ulica Sandomierska 16, 85-830 Bydgoszcz, Poland; ^2^Oftalmika Eye Hospital, Ulica Modrzewiowa 15, 85-631 Bydgoszcz, Poland; ^3^Department of Ophthalmology, Collegium Medicum, Nicolaus Copernicus University, Ulica Marii Curie-Skłodowskiej 9, 85-094 Bydgoszcz, Poland

## Abstract

*Purpose*. To evaluate the spatial distribution of photoreceptor inner and outer segment junction (IS/OS) reflectivity changes after successful vitrectomy for macula-off retinal detachment (PPV-mOFF) using spectral domain optical coherence tomography (SdOCT). *Methods*. Twenty eyes after successful PPV-mOFF were included in the study. During a mean follow-up period of 15.3 months, SdOCT was performed four times. To evaluate the IS/OS reflectivity a four-grade scale was used. *Results*. At the first follow-up visit the IS/OS had very similar reflectivity in entire length of the central scan with total average value of 1,05. At the second visit the most significant increase of the reflectivity was observed in temporal and nasal parafovea with average values of 2,17 and 2,22, respectively. The third region of increased reflectivity of an average value of 2,33 appeared during the third follow-up visit and was located in the foveola. At the last follow-up visit in entire central cross section the IS/OS reflectivity exceeded grade 2 reaching the highest average values in nasal and temporal parafovea and foveola. *Conclusions*. A gradual increase of the IS/OS reflectivity was observed in eyes after PPV-mOFF. The process is not random and starts independently in the peripheral and central part of the macula which may be attributed to the variable regenerative potential of cones and rods.

## 1. Introduction

In rhegmatogenous retinal detachment, the neurosensory retina becomes separated from the underlying retinal pigment epithelium (RPE), resulting in many biochemical and structural changes [1–4]. Morphological studies provide evidence that the most obvious detachment-induced damage within the neurosensory retina is shortening of the outer segments and gradual death of the photoreceptor cells [5–7]. Successful surgical treatment results in retinal reattachment to the RPE. This triggers a complex process of photoreceptors recovery that can lead to partial or even complete restoration of vision. The animal studies have demonstrated that healing is achieved by at least partial reconstruction of the outer segments of rods and cones [8]. The process is characterized by wide individual variation and depends strongly on the duration of detachment. Although complete restoration of the retinal structure has not been achieved, a modified version of the photoreceptor-RPE complex was established with significant variations in outer segment length and orientation. Studies based on animal models have also demonstrated that remodeling of the outer segments varies in intensity depending on the retinal area that is being remodeled [8].

The restoration of photoreceptor outer segments has been recently confirmed in vivo in humans using spectral domain optical coherence tomography (SdOCT) [9]. The hyperreflective band corresponding to the junction of photoreceptor inner and outer segments (IS/OS) was observed in 5% of eyes at one month and in 50% of eyes 6 months after surgery. Postoperative visual acuity correlated with a restored IS/OS line. However, the detailed dynamics of this process in relation to the particular macular areas is still unclear. The purpose of this study is to analyze topographically photoreceptor outer segments recovery process in patients after pars plana vitrectomy for retinal detachment using SdOCT.

## 2. Methods

The medical records of the patients who underwent pars plana vitrectomy for rhegmatogenous macula-off retinal detachment at the Department of Ophthalmology, Nicolaus Copernicus University, between January and June 2010 were reviewed. For further analysis records of 20 patients were selected. The inclusion criteria were as follows: successfully treated retinal detachment involved macula and two to four fundus quadrants, minimal number of four control visits during at least one year of follow-up. Exclusion criteria were as follows: high myopia, preexisting macular pathologies (e.g., macular hole or epiretinal membrane), and proliferative vitreoretinopathy stage C involving more than two hours. The eyes with the presence of any residual subretinal fluid in postoperative SdOCT exam were also excluded from the study. All patients underwent a pars plana vitrectomy with gas tamponade (SF6, C2F6, or C3F8). In each case perfluorocarbon liquid was used to perform internal drainage. All eyes except four were pseudophakic. One-step phacovitrectomy was performed in four phakic eyes. Mean follow-up period was 15,3 months. Patients' demographic data are summarized in Table 1. The study protocol was approved by an Ethics Committee of Nicolaus Copernicus University.

During follow-up visits patients underwent complete ophthalmic examination, including best-corrected visual acuity (BCVA) and fundus biomicroscopy. The first control visit was performed between the second and fourth weeks after surgery shortly after gas absorption. The second one took place between 4 and 12 weeks after surgery. The third visit was between 24 and 48 weeks and the fourth one between 48 and 72 weeks after surgery.

**Figure 1:**
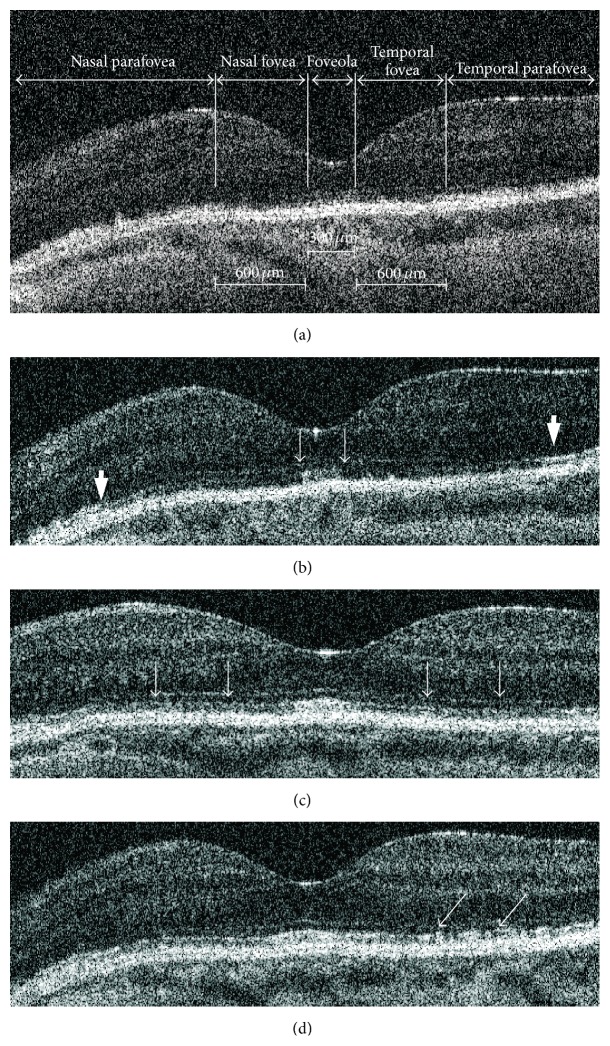
Restoration of normal IS/OS reflectivity after pars plana vitrectomy for macula-off rhegmatogenous retinal detachment in central SdOCT cross section. (a) Two weeks after surgery the IS/OS line is invisible in central part of the scan and has reduced reflectivity in periphery. (b) 10 weeks after surgery the IS/OS can be recognized in the entire scan and has reflectivity similar to normal one in foveola (thin arrows) and parafovea (thick arrows). (c) 44 weeks after surgery the IS/OS reflectivity increased in the area between foveola and parafovea (arrows). (d) 80 weeks after surgery only focal IS/OS disruptions can be observed (arrows).

SdOCT of the macula was performed during each visit (SOCT Copernicus, Optopol, Poland, or RS-3000 Nidek, Japan). Fifty-two to 64 horizontal scans of 6.0 mm length were performed, each consisting of 1024 lines. The reflectivity of the IS/OS line in central cross section running through foveolar center was evaluated independently by two OCT experts (JJK, BLS) according to the following subjective grading scale: grade 0: lack of the IS/OS line, grade 1: the reflectivity of the IS/OS line similar to the reflectivity of external limiting membrane (ELM), grade 2: the reflectivity of the IS/OS line higher than the reflectivity of ELM but lower then that of RPE (intermediate reflectivity), grade 3: the reflectivity of the IS/OS line similar to that of RPE. The SdOCT cross section was divided into five topographical sectors. The central one was foveola recognized as a flat retinal surface of 300 *μ*m in diameter in the center of foveal depression. The region located between the border of the foveola and the margin of foveal slope from nasal side was called nasal fovea and the corresponding region from temporal side was temporal fovea. Both of them have diameter of 600 *μ*m. Parts of the retina located outside fovea was named nasal and temporal parafovea (Figure 1). The grading was performed in each region independently.

Additionally, in eyes examined by the RS-3000 the en face reflectivity maps at the level of the IS/OS were created in automatic fashion using commercially available software. This facilitated evaluating the reflectivity of this layer throughout the entire examined area.

To evaluate statistical relationship between the grade of the IS/OS reflectivity and the retinal topography, the follow-up time, and the type of the used intraocular gas the chi-square test was used. Agreement between observers was evaluated by Bowker's test of symmetry. Correlation between BCVA and the IS/OS reflectivity was evaluated using Spearman's coefficient.

## 3. Results

Statistical analysis demonstrated good agreement between observers in evaluating the degree of the IS/OS reflectivity. The differences of the average grade in subjective scale were not statistically significant in any follow-up period. In all eyes the gradual process of at least partial restoration of normal IS/OS reflectivity was observed. The average IS/OS reflectivity grade within five topographical regions in the central SdOCT cross section in relation to the elapsed time since surgery is displayed in Table 2.

At the first follow-up visit (between the second and fourth week after surgery) the average reflectivity of the IS/OS line calculated in all 5 regions together was 1,05. Grade 0 was found in 38,8% of evaluated regions (Table 3). The IS/OS line had very similar reflectivity along the entire scan in all eyes except 9 in which the increase in reflectivity to grade 2 or 3 was observed in peripheral part of central cross section.

At the second follow-up visit (between the fourth and twelfth week after surgery) grade 0 appeared only in 3,33% of evaluated regions. Grade 2 was the most common finding observed in 40% of topographical locations. The average reflectivity measured in all 5 regions together increased to 1,74. The most significant increase in reflectivity was observed in temporal and nasal parafovea with average values of 2,17 and 2,22, respectively.

SdOCT exam performed during the third follow-up visit (between 24 and 48 weeks after surgery) showed further improvement in IS/OS appearance with the average reflectivity grade reaching value of 2,19. Grade 3 was the most common finding observed in 42,2% of evaluated locations. Grade 0 was not found in any location. When compared to the second follow-up visit, the third region, except peripheral parts of the scan, of average IS/OS reflectivity higher than grade 2 appeared. It was foveola with the average reflectivity of 2,33.

At the last follow-up visit (between 48 and 72 weeks after surgery) in all 5 regions of the central cross section the IS/OS reflectivity exceeded grade 2 reaching the highest average values in nasal and temporal parafovea and foveola. The average reflectivity calculated in all 5 regions together was 2,56. Grade 3 was observed in 61,3% of evaluated locations.

Statistical analysis confirmed that the grade of the IS/OS reflectivity during all follow-up visits except the first one was significantly related to the topographical regions of the central cross section. The process of restoration of the IS/OS reflectivity started in peripheral part of the central scan and was followed by the improvement of the IS/OS visibility in centrally located foveola with persistent areas of reduced reflectivity in nasal and temporal fovea.

**Figure 2:**
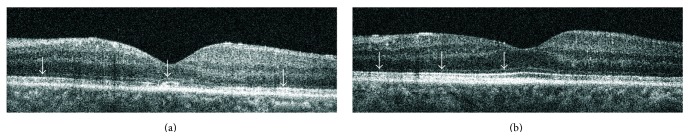
Asymmetrical increase of the IS/OS reflectivity after pars plana vitrectomy in central SdOCT cross section. (a) 4 weeks after surgery two areas of increased IS/OS reflectivity can be found in the foveola and parafovea (arrows). (b) 72 weeks after surgery area of normal IS/OS reflectivity appeared in foveola, parafovea, and nasal part of the fovea (arrows).

In majority of eyes the process of the IS/OS line restoration was symmetrical but in 30% of them the increase in the reflectivity was initially observed either in temporal or in nasal side of the scan. The opposite part of IS/OS band located between foveola and parafovea got normal reflectivity in the later follow-up or remained unchanged. Three eyes during final visit had highly reflective IS/OS layer in parafovea, foveola, and temporal part of the fovea with area of reduced reflectivity in nasal fovea. In one eye the normal IS/OS reflectivity returned in nasal part of the scan (Figure 2).

**Figure 3:**
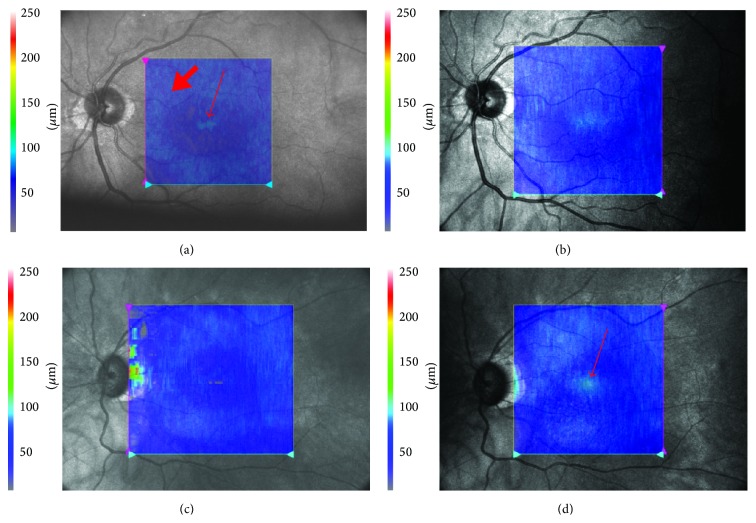
En face reflectivity maps at the level of the IS/OS layer. Maps were calculated for an area of 6 × 6 mm. The bright blue color depicts regions of normal IS/OS reflectivity. The dark blue color indicates areas of reduced IS/OS reflectivity. (a) Patient 1. Map calculated 9 months after surgery. Areas of normal reflectivity are visible in the peripheral part of the macula (thick arrow). A small, bright island is also visible in the center of the fovea (thin arrow). (b) Patient 1. 16 months after surgery. Areas of normal reflectivity have enlarged significantly. The superior macula and the central area of normal IS/OS have merged. (c) Patient 2. Seven months after surgery. An area of normal IS/OS is visible in the peripheral part of the macula (bright blue). (d) Patient 2. 14 months after surgery. A new area of normal reflectivity has appeared in the central fovea (arrow).

The above described changes are also visible on en face reflectivity maps (Figure 3). They show that the areas of normal IS/OS reflectivity initially appear in peripheral macula. Another small area of normal reflectivity is usually located in the center of the fovea. The maps made six months later reveal that the size of areas with increased reflectivity enlarged. As a result, peripheral and central areas of increased reflectivity approach each other, leaving only a small part of the macula with reduced reflectivity.

Visual acuity improved simultaneously with the increase of the IS/OS reflectivity. Low grade of reflectivity at the first follow-up visit was related to an average BCVA of 0.19. Eyes with partial recovery of IS/OS reflectivity at the second and third control visit had average BCVA of 0,37 and 0,49, respectively. The average BCVA during the final follow-up visit was 0,6. The correlation between BCVA and the grade of the IS/OS reflectivity was statistically significant (*p* < 0.0001; Spearman cc = 0,63). Statistical analysis revealed that the type of intraocular gas used during the surgery had no influence on the level of the IS/OS reflectivity.

## 4. Discussion

After a vitrectomy for macula-off rhegmatogenous retinal detachment that ends up with retinal reattachment the gradual change in reflectivity of the IS/OS line can be observed. Initially, the IS/OS line is usually invisible. It appears within the first weeks after surgery and gradually achieves normal reflectivity. This process typically starts independently in the peripheral and central macula and can last more than a year, often leading to a complete restoration of nearly normal IS/OS appearance.

Because of a significant difference in the refractive index present within photoreceptor cells, the IS/OS line is clearly visible on SdOCT in healthy eyes. It is noteworthy that recent data suggest better correlation of this hyperreflective line with photoreceptor ellipsoid zone rather than the junction between the inner and outer segments [10]. However, this does not change the clinical relevance of this line. Its unchanged appearance is often used in research as a marker of normal photoreceptor morphology. The abnormal IS/OS reflectivity was observed in patients after surgical treatment of macular holes, epiretinal membranes, and diabetic macular edema [11–13]. Published reports have also demonstrated photoreceptor defects in patients after surgically treated retinal detachment [14–16]. The defects were present in 76% to 83.3% of eyes within 3 to 7 months postoperatively, regardless of the performed procedure. In cases in which the mean time since the surgery was 23.1 months, photoreceptor defects were present in 53.3% of eyes [17]. Some authors point out that the IS/OS reflectivity can change over time, which may suggest some potential for tissue regeneration. This is related to progressive improvement of BCVA [18]. Similar changes have been described in the course of the natural healing process of such macular diseases as multiple evanescent white dot syndrome as well as in patients with surgically treated full-thickness and lamellar macular holes [19–21]. Shimoda et al. reported a reduction of the proportion of eyes with IS/OS abnormalities from 55% to 17% between the first and sixth postoperative months among patients in whom vitrectomy was performed because of retinal detachment, which may suggest gradual restoration of the outer photoreceptor segments [9].

The regeneration process of the retina has been described based on an animal model, which was used to analyze the microscopic changes that occur after retinal reattachment [8]. The results revealed that the length of the outer photoreceptor segments gradually increases depending on the duration of retinal detachment and time since reattachment. The strongest regeneration was observed in eyes with the shortest detachment time and the longest interval between the reattachment and the morphological examination. The authors also noted large discrepancies in the degree of regeneration among specific retinal regions. Moreover, the study demonstrated that the cone and rod regeneration processes differ. In cone regeneration, greater variability of outer segment length and generally lower regeneration potential were observed [8].

The topographic variability of changes in reflectivity patterns observed in our study may be associated with the differences between the cone and rod regenerative processes. The processes leading to increased reflectivity of the IS/OS start within the peripheral macula, where rods predominate, and gradually shift toward the center. At the same time, hyperreflective areas begin to appear within the central fovea, where only cones are present. These areas are initially small and tend to progress toward the peripheral macula. The observed regeneration processes are very individualized and do not always lead to complete IS/OS restoration. They can stop at any stage, resulting in abnormal reflectivity of the IS/OS junction and decreased visual acuity.

Our study shows that SdOCT can visualize gradually increasing IS/OS reflectivity in patients with retinal detachment that has been successfully treated with vitrectomy. This process is not random and follows different patterns within the central and peripheral macula which may be attributed to the variable regenerative potential of cones and rods.

## Figures and Tables

**Table 1:** Patients' demographic data.

Patients (*n*)	20
Sex (*n*)	
Women	11
Men	9
Mean age (years)	60,3
Lens status	
Pseudophakic (*n*)	16
Phakic (*n*)	4
Type of surgery (*n*)	
PPV + SF6	7
PPV + C2F6	7
PPV + C3F8	6
Mean follow-up (months)	15,3

**Table 2:** The average grade of the IS/OS reflectivity in SdOCT cross-sectional scan running through the foveolar center in relation to follow-up time.

	2–4 weeks after surgery	4–12 weeks after surgery	12–48 weeks after surgery	48–72 weeks after surgery
Nasal parafovea	1,29	2,22	2,61	2,87
Nasal fovea	0,94	1,33	1,67	2,13
Foveola	0,82	1,72	2,33	2,73
Temporal fovea	0,88	1,28	1,83	2,33
Temporal parafovea	1,29	2,17	2,50	2,73

**Table 3:** The percentage distribution of the IS/OS reflectivity in SdOCT cross-sectional scan running through the foveolar center in relation to follow-up time.

	2–4 weeks after surgery	4–12 weeks after surgery	12–48 weeks after surgery	48–72 weeks after surgery
Grade 0	38,82%	3,33%	0,0	0,0
Grade 1	25,88%	37,78%	23,33%	5,33%
Grade 2	27,06%	40,00%	34,44%	33,33%
Grade 3	8,24%	18,89%	42,22%	61,33%
